# Patient Similarity in Prediction Models Based on Health Data: A Scoping Review

**DOI:** 10.2196/medinform.6730

**Published:** 2017-03-03

**Authors:** Anis Sharafoddini, Joel A Dubin, Joon Lee

**Affiliations:** ^1^ Health Data Science Lab School of Public Health and Health Systems University of Waterloo Waterloo, ON Canada; ^2^ Department of Statistics and Actuarial Science University of Waterloo Waterloo, ON Canada

**Keywords:** patient similarity, predictive modeling, health data, medical records, electronic health records, personalized medicine, data-driven prediction, review

## Abstract

**Background:**

Physicians and health policy makers are required to make predictions during their decision making in various medical problems. Many advances have been made in predictive modeling toward outcome prediction, but these innovations target an average patient and are insufficiently adjustable for individual patients. One developing idea in this field is individualized predictive analytics based on patient similarity. The goal of this approach is to identify patients who are similar to an index patient and derive insights from the records of similar patients to provide personalized predictions..

**Objective:**

The aim is to summarize and review published studies describing computer-based approaches for predicting patients’ future health status based on health data and patient similarity, identify gaps, and provide a starting point for related future research.

**Methods:**

The method involved (1) conducting the review by performing automated searches in Scopus, PubMed, and ISI Web of Science, selecting relevant studies by first screening titles and abstracts then analyzing full-texts, and (2) documenting by extracting publication details and information on context, predictors, missing data, modeling algorithm, outcome, and evaluation methods into a matrix table, synthesizing data, and reporting results.

**Results:**

After duplicate removal, 1339 articles were screened in abstracts and titles and 67 were selected for full-text review. In total, 22 articles met the inclusion criteria. Within included articles, hospitals were the main source of data (n=10). Cardiovascular disease (n=7) and diabetes (n=4) were the dominant patient diseases. Most studies (n=18) used neighborhood-based approaches in devising prediction models. Two studies showed that patient similarity-based modeling outperformed population-based predictive methods.

**Conclusions:**

Interest in patient similarity-based predictive modeling for diagnosis and prognosis has been growing. In addition to raw/coded health data, wavelet transform and term frequency-inverse document frequency methods were employed to extract predictors. Selecting predictors with potential to highlight special cases and defining new patient similarity metrics were among the gaps identified in the existing literature that provide starting points for future work. Patient status prediction models based on patient similarity and health data offer exciting potential for personalizing and ultimately improving health care, leading to better patient outcomes.

## Introduction

Medicine is largely reactive—a disease is treated only after it is observed [1]. However, a move toward proactive medicine has been initiated by advances in technologies for analyzing the nature of a disease or estimating individual susceptibility to disease [2]. Moreover, a sharp increase in electronic health record (EHR) adoption has facilitated the move toward proactive medicine, which will hopefully lead to improved care and better patient outcomes. However, it is challenging for clinicians to examine and derive insights from multidimensional, large-scale EHR data. One pathway to proactive medicine employs predictive analytics to accurately derive insights from EHR data to predict disease progression. Predictive analytics, by employing EHRs, can also lead to personalized decision making based on the unique characteristics of a given patient [3].

Many studies have analyzed large populations to answer a wide range of health-related questions, including the study that developed Acute Physiology and Chronic Health Evaluation II (APACHE-II) [4]. These studies often provide statistically rigorous results for an average patient but are also expensive, time-consuming, and prone to selection bias [1]. Moreover, one of the major challenges for population-based studies is comorbidity, which limits generalizing a study to many patients [5,6]. Typically, these studies provide “the average best choice” [3]. Therefore, physicians cannot solely rely on the evidence from such population-based studies when facing a patient with conditions that deviate from the average.

One developing idea in this field is personalized predictive modeling based on patient similarity. The goal of this approach is to identify patients who are similar to an index patient and derive insights from the records of similar patients to provide personalized predictions. Employing patient similarity helps identify a precision cohort for an index patient, which will then be used to train a personalized model. Compared to conventional models trained on all patients, this approach has the potential to provide customized prediction. This approach has been widely used for personalized predictions in other fields, including music [7], movies [8], and sales pricing [9], and is referred to as *collaborative filtering* [10]. It can potentially be employed to manage a real-world patient with a complex health status and comorbidity profile. Patient similarity analytics also has the potential to assess the similarity between an index patient and trial population in conventional studies and help clinicians choose the most appropriate clinical trial [11].

Although the concept of patient similarity is not new—blood typing has been used for blood transfusion for more than a century [12]—advanced application of patient similarity is missing in the new era of data-driven medicine. The online PatientsLikeMe website provides a patient-reported database, where an index patient can find a cohort of similar patients and explore their data including symptoms, treatments, and tests [13]. Although PatientsLikeMe received the Drug Information Association 2014 President’s Award for Outstanding Achievements in World Health, the full potential of patient similarity, especially in predictive modeling, has not been uncovered. Although there have been some attempts to embed patient similarity in health predictive modeling, a comprehensive picture of patient health predictive analytics based on health data (including EHRs) and patient similarity is lacking in the literature. The objectives of this paper are to provide an analysis and summary of the studies on patient health prediction models based on health data and patient similarity, identify any gaps in this area, and suggest ideas for future work. Overall, this review aims to address the following three research questions:

In which context (applications) have patient health prediction models based on health data and patient similarity been used?

Which modeling techniques have been considered in the literature?

How do patient similarity-based models affect health predictions in comparison to conventional models?

We hope the results could also contribute to the broad field of case-based reasoning (CBR)—with the core component of similarity assessment—to meet the challenges in medical applications [14].

## Methods

A systematic search approach in line with guidelines of Kitchenham et al [15] was taken to review and analyze the literature on patient similarity in health prediction models based on health data. However, this paper does not aim to report the performance of particular models and identify the best model because various health data types and performance measures are possible.

### Inclusion and Exclusion Criteria

Studies included in this review had to be journal articles or conference proceedings written in English. They had to focus on prediction in the health domain, devise a model for prediction, embed explicit patient similarity analytics, and utilize health data for training their model. Studies were excluded if (1) they entirely relied on human input for predictions or similarity assessment, (2) the model was tested on seen data—the part of the data used for training the algorithm, and (3) the algorithm was trained using only genomic data. If the same study appeared in multiple publications, only the most comprehensive and latest version was included.

### Paper Selection

The literature search was finalized in December 2015. Scopus, PubMed, and ISI Web of Science, all databases covering health-related publications, were searched for peer-reviewed studies with keywords related to “prediction,” “health data,” and “patient similarity.” The search strings used in each of these search engines are given in Multimedia Appendix 1. After removal of duplicates, the title and abstract of each identified article were screened. The remaining articles were further examined in full text to finalize the set of included articles.

### Data Extraction and Analysis

Data from included articles were extracted into a matrix table and analyzed with respect to the following criterion: publication information, context, predictors (or features), missing data, modeling algorithms, performance measures, and outcomes. The context was further examined from two points of view: data source and application area. The employed patient similarity-based modeling algorithms were also synthesized in three categories: neighborhood-based, clustering-based, and other algorithms, with the majority falling in the first category. Because measuring predictive performance is essential to model development (model selection/model tuning)—and can also be used to compare a given model with other methods (performance estimation)—evaluation metrics along with validation techniques used in the reviewed studies were also extracted.

## Results

**Figure 1:**
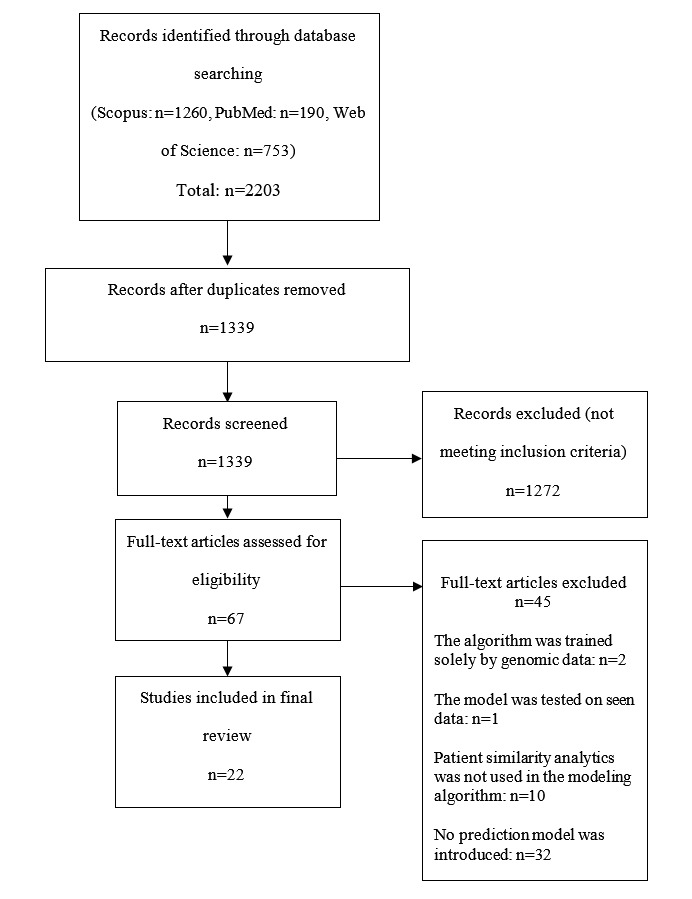
Flow diagram of article selection procedure.

A total of 22 articles were included in the review (Figure 1). Tables 1 and 2 summarize the data extracted from input data/predictors and outcome perspectives, respectively.

**Table 1:** Summary of the reviewed articles in terms of data type, data origin, number of predictors, and number of instances (N=22).

Authors	Data type	Data origin^a^	Predictors, n^b^	Instances, n^c^
	Cross-sectional			
Jurisica et al [16]	Cross-sectional	NR	55	788
Bobrowski [17]	Cross-sectional	The Gastroenterological Clinic of the Institute of Food and Feeding in Warsaw [18]—a database consisting of hepatological patient data	40	511
Park et al [19]	Cross-sectional	UCI repository [20]-Dermatology	35	350
	Cross-sectional	UCI repository [20]-Heart Disease: Cleveland Clinic;	13	270
	Cross-sectional	UCI repository[20]-Breast Cancer Wisconsin: University of Wisconsin Hospital and Clinics	31	560
	Cross-sectional	UCI repository[20]-Pima Indians Diabetes: NR	8	760
	Cross-sectional	UCI repository[20]-Liver Disorders: BUPA Medical Research	7	340
Saeed et al [21]	Longitudinal	MIMIC-II [22]-The Multiparameter Intelligent Monitoring in Intensive Care at Boston’s Beth Israel Deaconess Medical Center	50	377
Chattopadhyay et al [23]	Cross-sectional	Hospital-history of suicidal attempts and committed suicides collected from hospital records	15	50
Sun et al [24]	Longitudinal	MIMIC-II [22]-The Multiparameter Intelligent Monitoring in Intensive Care at Boston’s Beth Israel Deaconess Medical Center	50	74
Sun et al [25]	Longitudinal	MIMIC-II [22]-The Multiparameter Intelligent Monitoring in Intensive Care at Boston’s Beth Israel Deaconess Medical Center	10	1500
David et al [26]	Cross-sectional	Laboratory results generated by two Beckman-Coulter Gen-S analyzers at an acute care facility in Brooklyn	NR	4900
Houeland [27]	Cross-sectional	A dataset focused on palliative care for cancer patients	55	1486
Wang et al [28]	Cross-sectional	UCI repository [20]-Breast Cancer Wisconsin: University of Wisconsin Hospital and Clinics	31	560
	Cross-sectional	UCI repository [20]-Pima Indians diabetes	8	760
	Cross-sectional	A real-world EHR data warehouse of a health network consisting of data from 135K patients over a year	NR	135K
Wang et al [29]	Cross-sectional	A real-world EHR data warehouse of a health network consisting of data from 135K patients over a year	2388	3946
Campillo-Gimenez et al [30]	Cross-sectional	French Renal Epidemiology and Information Network (REIN) registry [31]	19	1137
Gottlieb et al [32]	Cross-sectional and longitudinal	Hospital dataset-Stanford Medical Center, USA	16	9974
	Cross-sectional and longitudinal	Hospital dataset-Rabin Medical Center, Israel	16	5513
Lowsky et al [33]	Cross-sectional	A dataset by the United States Renal Data System (USRDS) consisting of all kidney transplant procedures from 1969 to 1999	13	51,088
Hielscher et al [34]	Cross-sectional	The Study of Health in Pomerania (SHIP) [35]-a dataset consisting of a comprehensive examination program including but not limited to ultrasound tests and laboratory analysis	65/57	578
Zhang et al [36]	Longitudinal	A 3-year longitudinal EHR data of 110,157 patients	NR	1219
Henriques et al [37]	Longitudinal	myHeart home telemonitoring study [38]-daily physiological records including blood pressure, respiration rate, heart rate, and body weight	NR	41
Lee et al [39]	Cross-sectional and longitudinal	MIMIC-II [22]-The Multiparameter Intelligent Monitoring in Intensive Care at Boston’s Beth Israel Deaconess Medical Center	76	17,152
Ng et al [40]	Cross-sectional and longitudinal	A longitudinal medical claims database consisting of data from over 300,000 patients during four years	8500	15038
Panahiazar et al [41]	Cross-sectional	The Mayo Clinic	33	1386
Wang [42]	Cross-sectional	UCI repository [20]-Breast Cancer Wisconsin: University of Wisconsin Hospital and Clinics	31	560
	Cross-sectional	UCI repository[20]-Pima Indians diabetes	8	760
	Cross-sectional	A real-world EHR data warehouse	NR	135K
Wang et al [43]	Cross-sectional	A real-world EHR data warehouse	127	3946

^a^ NR: not reported.

^b^ Predictors: the total number of predictors.

^c^ Instances: the total number of data points used in each study including the training and test.

**Table 2:** Summary of reviewed articles in terms of outcome, evaluation metrics, and comparing methods (N=22).

Authors	Outcome^a^	Evaluation metrics^b^	Compared against^c^
Jurisica et al [16]	Suggesting hormonal therapy (day of human chorionic gonadotrophin administration and the number of ampoules of human menopausal gonadotrophin) after in vitro fertilization and predicting pregnancy outcome (pregnancy, abortion, ectopic pregnancy, and ovarian hyperstimulation syndrome)	Accuracy	NR
Bobrowski [17]	Four types of liver disease (cirrhosis hepatis biliaris primaria, cirrhosis hepatis decompensata, hepatitis chronica activa, and hepatitis chronica steatosis)	Accuracy	Classic *k*-NN (*k*=10)
Park et al [19]	(1) Six types of dermatology diseases (psoriasis, seborrheic dermatitis, lichen planus, pityriasis rosea, chronic dermatitis, pityriasis rubra pilaris); (2) diagnosis of heart disease (angiographic disease status); (3) diagnosis of a breast tumor as malignant or benign; (4) diagnosis of diabetes; (5) diagnosis of liver disorder	Accuracy; sensitivity; specificity	LR; C5.0; CART; neural network; conventional CBR (*k*=5) with five neighbors
Saeed et al [21]	Hemodynamic stability or instability of an episode	Sensitivity; positive predictive value	NR
Chattopadhyay et al [23]	Suicidal risk levels (level 1: suicidal plans or thoughts; level 2: single suicidal attempt; level 3: multiple suicidal attempts)	NR	NR
Sun et al [24]	Occurrence of acute hypotensive episode within the forecast window of an hour	Accuracy	Human expert’s idea based on the Euclidean [44]; *k*-NN over low-dimensional space after applying PCA
Sun et al [25]	Occurrence of acute hypotensive episode within the forecast window of an hour	Accuracy	Human expert’s idea based on the Euclidean [44]; *k*-NN over low-dimensional space after applying PCA
David et al [26]	Seven disease diagnoses (microcytic anemia, normocytic anemia, mild SIRS, thrombocytopenia, leukocytopenia, moderate/severe SIRS, normal)	Accuracy	Human expert’s idea
Houeland [27]	Pain levels	Error rate (1-accuracy).	Random retrieval; *k*-NN (k=1) with the Euclidian distance; random forest
Wang et al [28]	(1) Diagnosis of a breast tumor as malignant or benign; (2) diagnosis of diabetes; (3) diagnosis of dementia without complications (HCC352) or diabetes with no or unspecified complications (HCC019)	Accuracy; sensitivity; precision; F-measure	PCA; LDA [45]; LSDA [45]; LSML [24]
Wang et al [29]	Diagnosis of CHF 6 months later	Accuracy; sensitivity; precision; F-measure	LLE; LE; PCA; Euclidean distance.
Campillo-Gimenez et al [30]	Registration on the renal transplant waiting list: yes/no	ROC curve	*k*-NN; LR; *k*-NN with weighted predictors; *k*-NN with weighted patients
Gottlieb et al [32]	Patient discharge diagnosis *ICD* codes	ROC curve; F-measure	NR
Lowsky et al [33]	Graft survival probability	IPEC	Cox model; RSF [46]
Hielscher et al [34]	Three levels of liver fat concentration measured by magnetic resonance tomography: (1) fat concentration <10%; (2) fat concentration of 10%-25%; (3) fat concentration ≥25%	Accuracy; sensitivity; specificity	Multiple variants of the *k*-NN: majority voting; weighted voting; with/without predictor selection	
Zhang et al [36]	Four effective drugs for hypercholesterolemia treatment: atorvastatin, lovastatin, pravastatin, and simvastatin	ROC curve	Patient similarity; patient similarity with drug structure similarity; patient similarity with drug target similarity	
Henriques et al [37]	Early detection of heart failure: decompensation or normal condition	Sensitivity; specificity; F-measure; G-measure	Coefficients’ distance; linear correlation of signals; Euclidean distance
Lee et al [39]	30-day in-hospital mortality	Area under ROC curve; area under precision-recall curve	Population-based and personalized versions of: majority vote; LR; DT
Ng et al [40]	The risk of diabetes disease onset	ROC curve	Global LR; *k*-NN; patient similarity-based LR with Euclidean distance
Panahiazar et al [41]	Medication plans for heart-failure patients (angiotensin-converting enzyme, angiotensin receptor blockers, β-adrenoceptor antagonists, statins, and calcium channel blocker)	Sensitivity; specificity; F-measure; accuracy	K-means; hierarchical clustering	
Wang [42]	(1) Diagnosis of a breast tumor as malignant or benign; (2) diagnosis of diabetes; (3) occurrence of CHF within 6 months	Precision; F-measure; sensitivity; accuracy	*kd*-tree; PCA- *kd*-tree; ball-tree; spectral-tree.	
Wang et al [43]	Occurrence of CHF within 6 months	Precision; F-measure; sensitivity; accuracy	PCA; Laplacian regularized metric learning [47]; LLE [48]; LSR; LSML [24]

^a^ CHF: congestive heart failure; *ICD*: *International Classification of Diseases*.

^b^ IPEC: integrated prediction error curve ; NR: not reported; ROC: receiver operating characteristic: SIRS: systemic inflammatory response syndrome.

^c^ CART: classification and regression tree; CBR: case-based reasoning; DT: decision tree; *k*-NN: *k-* nearest neighbor; *kd*-tree: *k* dimensional tree; LDA: linear discriminant analysis; LE: Laplacian embedding; LLE: locally linear embedding; LR: logistic regression; LSDA: locality sensitive discriminant analysis; LSML: locally supervised metric learning; LSR: local spline regression; NR: not reported; PCA: principal component analysis; RSF: random survival forest.

### Publication Information

**Figure 2:**
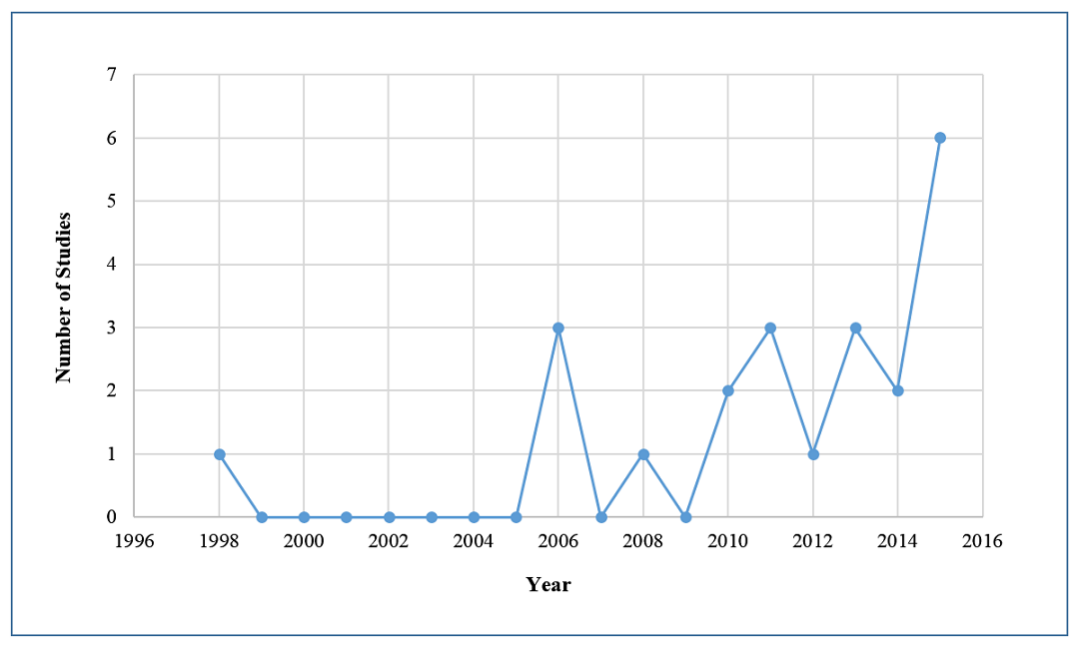
Distribution of publications by year.

The level of interest could be gauged by the increase in publication on this topic in recent years (Figure 2). Fifteen studies of 22 were journal publications [16,17,19,21,23,25,26,30,32,33,36,37,39,40,42,43] and seven were conference articles [24,27-29,34,41].

### The Context

**Figure 3:**
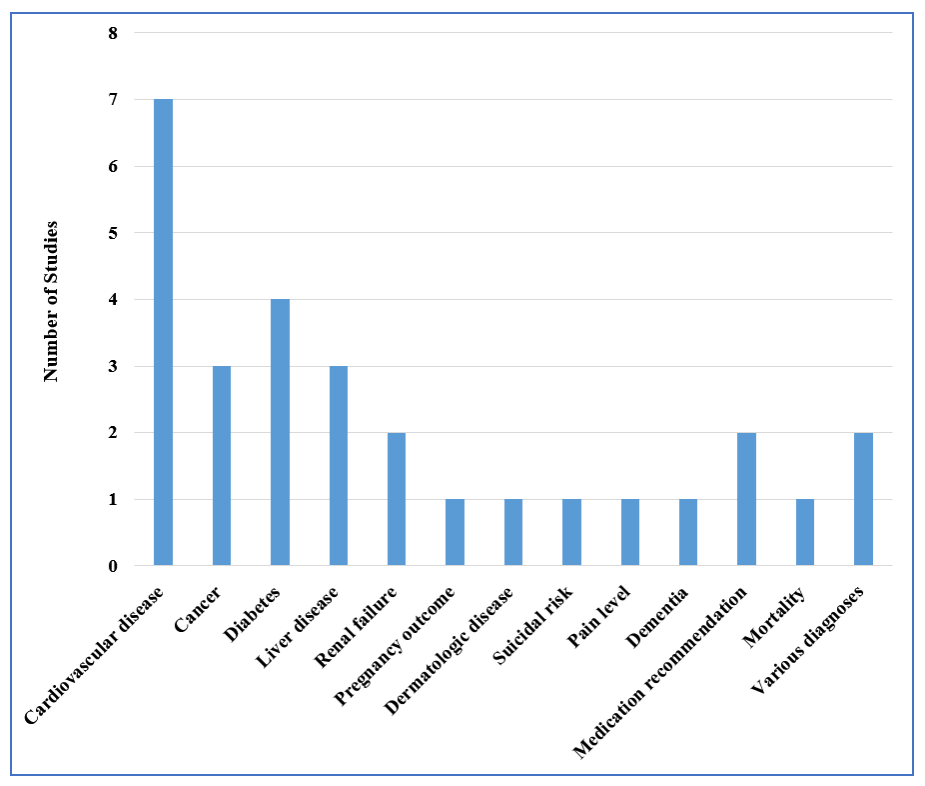
Focused application areas of studies. Some studies featured more than a single application area and were counted more than once.

Although a considerable number of articles did not clearly state the source of data—some articles used more than one dataset—hospitals were named (10/22); within hospitals, intensive care units (ICUs) were the main sources of data (5/10). In addition, one study [19] used data from a research center and another study [37] utilized telemonitoring data, also known as wearable-based remote patient monitoring data. From the application area perspective, chronic diseases were the most prevalent context. For detailed distributions, refer to Figure 3.

### The Predictors

Raw health data can be in various formats, including narrative/textual data (eg, history of a present illness), numerical measurements (eg, laboratory results, vital signs, and measurements), recorded signals (eg, electrocardiograms), and pictures (eg, radiologic images). Numerical measurements and recorded signals were the format used most in the reviewed articles. Three main approaches were used for extracting predictors from raw health data. First, for some variables, including age and gender, the exact/coded value was used as a predictor. Second, in articles employing recorded signals and/or longitudinal numerical measurements, numeric variables, including wavelet coefficients, minimums, maximums, means, and variances, were extracted from within particular time windows [24,25,37,39,41]. Third, three studies [21,29,43] employed the term frequency-inverse document frequency (TF-IDF) technique from text mining to produce predictors for their model.

Although predictor extraction affects the performance of the model [24], one of the challenging tasks in patient similarity-based predictive modeling is identifying the most relevant and important patient characteristics for patient similarity assessment. Patient similarity assessment is generally defined as investigating the similarity of patients’ data in terms of their symptoms, comorbidities, demographics, and treatments, but there is no predefined list of predictors to be considered. Most of the studies proposed an arbitrary list of predictors or limited their work to the available predictors, but selected predictors must be representative of patient’s condition in each particular application. Two studies [26,30] employed weighting schemes to adjust the importance of the predictors based on the outcome. One study [34] showed that predictors selected by a correlation-based feature algorithm could vary according to gender. Although feature selection methods can help with predictor selection, selected predictors may not be the most appropriate ones for each individual patient because they are derived from general analysis of the population. One study [40] showed that a group of similar patients has a similar set of predictors, but the predictors’ importance was different between individuals. Two studies [29,43] suggested utilizing expert knowledge on the similarity of cases to implicitly consider case-specific predictors. One study [16] proposed a context-based similarity metric in which an expert determined a set of predictors and their allowable values for patient similarity assessment.

### Missing Data

One common challenge in using health data in predictive analytics is missing data. Most of the modeling techniques cannot handle an incomplete data matrix. Nevertheless, all studies that mentioned this challenge [19,23,25,30,32,39,41], except for two [23,25], simply excluded patients with incomplete data. In Chattopadhyay et al’s study [23], missing values were replaced with the most common value of the corresponding predictor. Sun et al [25] evaluated two methods in overcoming missingness: replacing the missing value with the mean of the sensor measurements within a time window or imputing based on the correlations among multiple sensors using linear regression models. The latter method consistently performed better than the former. Three studies [21,29,43] that mapped EHRs to TF-IDF space handled the missing data challenge indirectly. Other studies did not discuss missing data.

### The Modeling Algorithms

#### Neighborhood-Based Algorithms

The neighborhood-based algorithms indicate studies in which a group of patients similar to an index patient is retrieved and a prediction is produced by a model trained on similar patients’ data. This category is comparable to memory-based techniques in collaborative filtering [10]. Various types of similarity metrics can describe the similarity between patients. Studies in this category [16,17,19,21,23-26,28,30,33,34,37,39,40,43,47] were organized based on the type of similarity metric they employed for calculating patient similarity.

#### Distance-Based Similarity Metrics

Twelve studies (of 18) used various types of distance-based similarity. One study [23] utilized the sum of absolute distances for each predictor to retrieve a cohort of similar patients and find the closest class to a new patient.

Five studies [17,19,26,34,43] utilized the Euclidean distance. Bobrowski [17] designed a linear transformation by solving a convex optimization problem to maximize between-class distances and minimize in-class distances. In this study, the *k*-nearest neighbor (*k*-NN) method on the transformed data outperformed the classical *k*-NN algorithm. Park et al [19] investigated the optimum number of neighbors for each patient. In this study, a grid search found a cut-off probability based on the distribution of pairwise distances to define a distance threshold. This method outperformed several conventional machine learning algorithms, including logistic regression (LR), C5.0 decision tree (DT), classification and regression tree, neural network, and conventional CBR.

David et al [26] employed the Euclidean distance on weighted predictors to select neighbors for an index patient. Although their method strongly agreed with a human reviewer, no comparison with other methods was reported. Hielscher et al [34] suggested the idea of subgrouping the training set based on gender and then applying a *k*-NN method. This study showed that using a predictor selection algorithm can reduce the dimension of the predictor space and improve performance. Furthermore, the results demonstrated that only a few of the predictors with highest predictive power within each subgroup are common, thus highlighting the efficiency of subgrouping a population, then considering customized predictors for each subgroup.

Six studies utilized the Mahalanobis distance [24,25,28,29,33,40]. Sun et al [24] defined a Mahalanobis distance by solving an optimization problem aimed at minimizing the within-class squared distances and maximizing between-class squared distances. A sensitivity analysis of the parameter *k—* number of neighbors *—* revealed that small *k* resulted in lower classification error, confirming the idea of using local information. The proposed metric outperformed the Euclidean distance. As an extension of a previous study [12], Sun et al [25] trained a linear regression model based on a least squared error fitting technique on the retrieved data. The proposed method outperformed the previous method and *k*-NN with Euclidean distance on a lower dimensional space mapped by linear discriminant analysis.

Wang et al [28] focused on integrating multiple patient similarity metrics learned independently without sharing the training datasets. In combining the metrics, various degrees of importance were considered for each individual Mahalanobis metric. The proposed method outperformed all compared methods and improved accuracy even when some individual metrics were biased. Building on that study, Wang et al [29] proposed a new algorithm in which human experts’ ideas could be embedded. To incorporate expert knowledge, two matrices were defined by an expert: a similarity matrix and dissimilarity matrix. The proposed method outperformed *k*-NN with the Euclidean distance in the original feature space and low-dimensional spaces derived by PCA, locally linear embedding and Laplacian embedding.

Wang [43] then proposed a two-term objective function for Mahalanobis distance learning: a part based on human experts’ knowledge (following the same procedure as in the previous study) and a part based on available historical data. The proposed online distance metric learning method outperformed locally supervised metric learning [24]. In addition, the results showed that the performance increased from 20 to 200 neighbors, but decreased after 200. This result supports the advantage of using local neighborhood data.

Lowsky et al [33] proposed a neighborhood-based survival probability prediction model based on a Mahalanobis distance and constructed a weighted Kaplan-Meier survival curve on the basis of retrieved similar cases. Although their method did not show consistent advantage over the Cox model on the original dataset, its performance improved as the proportional hazards violation was highlighted on the simulated datasets.

Ng et al [40] compared personalized predictive modeling and population-based predictive models. The proposed algorithm made predictions using Mahalanobis and an LR model. Clustering analysis of risk factors revealed that patients with similar risk factors were grouped together, whereas patients with different risk factors were distributed in groups far apart in the cluster tree. Furthermore, a large number of risk factors were not captured by the population-based model, whereas personalized models highlighted them.

#### Correlation-Based Similarity Metrics

Saeed et al [21] utilized a correlation coefficient to retrieve the *k* most similar patients. This correlation coefficient measured the extent of the linear correlation between two data points. The proposed algorithm was not benchmarked against other methods.

#### Cosine-Similarity Metrics

Lee et al [39] examined the hypothesis that predictive modeling based on patient similarity analytics can outperform conventional predictive modeling in which all available patient data are analyzed. Their study employed cosine patient similarity and focused on characterizing neighborhood size and model performance. Results confirmed that patient similarity analytics can outperform not only population-based models but also well-known clinical scoring systems. Moreover, a reasonably small and homogenous neighborhood improved predictive performance; however, a very small neighborhood compromised performance due to small sample size effects.

#### Other Similarity Metrics

Four studies used other similarity metrics. One of the earliest proposed methods [16] retrieved patients based on the context defined by a user. A context was defined as a set of predictors and had allowable values for these predictors in the retrieval task. Houeland [27] proposed a combination of a Euclidean distance and a tree-based distance. Each case in the training set was stored with its associated terminal node for every tree in a forest of randomly grown trees. For a new patient, half of the most similar patients in the training set were retrieved based on Euclidian distance. Then, two patients were considered to be more similar if they shared the same terminal node assignments for a higher number of trees. The proposed method outperformed conventional random forest and *k*-NN with the Euclidean distance.

Campillo-Gimenez et al [30] employed an exclusive OR-based patient similarity metric with an LR model. This method outperformed compared methods, including population-based LR, and performed well, after randomly generated predictors were added to the relevant predictors. Henriques et al [37] utilized a similarity metric based on the signs of Haar wavelet coefficients derived from telemonitoring data. A metric based on the coefficients’ signs outperformed similarity metrics based on the coefficients’ distances, Euclidian distance, and linear correlation of the actual data points.

#### Cluster-Based Algorithms

Cluster-based algorithms group patients in a training set based on their profiles and relationships. Therefore, a new patient is assigned to a predefined cluster based on his/her similarity to each cluster. These methods have a trade-off between prediction performance and scalability for large datasets. Only one study [41] employed supervised and unsupervised clustering approaches with a Mahalanobis distance in recommending a medication to a heart-failure patient. Then, the most frequently prescribed medication in the most similar cluster was selected for the index patient. The proposed supervised clustering outperformed hierarchical clustering and *k*-means.

#### Other Algorithms

Gottlieb et al [32] focused on associations between hospitalization data and discharge diagnoses, considering eight similarity metrics between hospitalization data and two similarity measures for *International Classification of Diseases* codes. Then, they combined these measures into 16 hospitalization-discharge code associations. For a new patient’s hospitalization data, the score of a potential discharge code was calculated by considering the similarity to the known discharge code-hospitalizations’ associations, and then an LR classifier was trained to distinguish true associations (of medical history with diagnosis) from false ones. Using various similarity metrics helped overcome the limitations of using only one particular similarity metric—using just one similarity metric for all predictors may miss information relevant to prediction [49].

Zhang et al [36] augmented patient similarity analytics with drug similarity analytics and proposed an algorithm for personalized drug recommendations in hypercholesterolemia treatment. Based on the Jaccard similarity metric in their label propagation algorithm, they defined three sets of similarities: (1) patient-patient, (2) drug-drug, and (3) patient-drug. This study suggested that combining patient similarity with drug similarity can help achieve personalized medicine.

Wang [42] proposed an adaptive semisupervised recursive tree partitioning (ART) approach to reduce the computational burden of pairwise patient similarity calculations. This algorithm can also leverage expert knowledge. The algorithm constructs a tree used to index patient profiles and then rapidly retrieve the nearest neighbors to a new patient. The ART series methods generally performed better than compared methods.

### Outcomes

The outcomes of prediction models normally take six forms: continuous, binary, categorical (but not ordered), ordinal, count, and survival. The studies reviewed targeted continuous outcomes [16], such as hormonal therapy dosage; binary outcomes [19,21,24,25,28-30,37,39,42,43], such as disease diagnosis or patient death; categorical outcomes [17,19,26,32,36,41], such as multiple-disease diagnosis; and ordinal outcomes [23,27,34,40], such as the grade of an illness. One study also aimed to predict a survival outcome [34] (ie, the prediction of the time to an event of interest) [50]. No study had a count outcome, which is a nonnegative integer value derived from counting rather than grading.

### Evaluation Metrics and Validation Techniques

#### Evaluation Metrics

Evaluation metrics are widely used to tune the parameters of a model and compare the model with other methods.

#### Evaluation Metrics Based on a Confusion Matrix

A confusion matrix is a cross-tabulation representation of observed and predicted classes. Various evaluation metrics extracted from a confusion matrix—including accuracy, sensitivity, specificity, F-measure, G-measure, precision, and positive predictive value—were used in the included articles.

#### Receiver Operating Characteristic Curve

Five articles [30,32,36,39,40] used the receiver operating characteristic (ROC) curve, and one [39] used the precision-recall curve in combination with the ROC curve to overcome the optimistic estimate of ROC curves in the presence of imbalanced data—where class distribution is not approximately uniform among the classes.

#### Measures Based on Model Residuals

When a model generates a continuous outcome, a common performance measure is the mean squared error. This metric is based on model residuals, which are the difference between the observed and predicted responses, and can be calculated by taking the average of squared model residuals. One study [25] used a relative error rate and another [33] used integrated prediction error curve, a time-invariant measure that calculates the weighted quadratic difference of prediction and observed survival outcome.

#### Validation Techniques

Validation techniques can generally be grouped into two categories: internal and external [50].

##### Internal Validation Techniques

Internal validation techniques randomly split the available dataset into two parts using various approaches: a training set and a test set. Seven studies [21,23,25,26,28,30,51] used various ratios for this splitting. Eight studies [19,32,34,36,39-42] employed a *k*-fold cross-validation technique and five studies [16,17,24,27,37] used leave-one-out.

##### External Validation Techniques

External validation means assessing the performance of the prediction model in other scenarios or settings (eg, assessing the geographic or temporal transportability of the model). Only one study [33] used temporal validation for assessing their model’s performance. External validation better evaluates generalization of a model to new patients.

## Discussion

Over the period of 1989 to 2015, we found 22 articles that focused on patient similarity in predictive modeling using EHR data, with an increase in the number of these studies over time. Overall, three main approaches were employed in these studies to leverage patient similarity: neighborhood-based modeling, clustering modeling, and other algorithms. This section discusses the results from this review study to address the research questions then identifies gaps and future research directions.

### Predictive Modeling Based on Patient Similarity and Health Data Context

This study showed that patient similarity-based predictive modeling has been widely used on hospital data, which sheds light on the need for patient similarity-based predictive modeling in tackling big data. In addition, further analysis revealed that ICUs are the central focus in hospitals. ICUs treat patients with severe and life-threatening illnesses that require continuous monitoring. Thus, ICU patients are surrounded by equipment that constantly generates a large amount of data. However, this large volume usually overwhelms clinicians and highlights the need for a computerized system. In addition, the critical health status of the patients in ICUs requires more proactive (rather than reactive), precise, and personalized care. Therefore, ICUs are a suitable environment for personalized prediction models.

Furthermore, chronic disease prognosis was one of the common application areas for personalized predictive modeling. Such analytics can help in improving patient health status if used in planning new therapies or interventions to prevent further complications. Patient similarity analytics can also be used for predicting a patient’s risk of developing further complications or disease. In particular, patient similarity analytics can overcome the challenge of comorbidities in chronic disease risk stratification and provide customized plans for a given patient. It is worth mentioning that cardiovascular diseases and diabetes were common application domains among the reviewed studies.

### Modeling Techniques

Most of the studies focused on neighborhood-based modeling. These models are easy to implement and they typically perform well. However, their performance depends greatly on the chosen patient similarity metric. Although there are a variety of similarity metrics in data mining [52], distance-based similarity metrics were the most popular in the reviewed studies. These methods are also constrained by their limited scalability for big data. Although Lee et al [39] suggested that computational load can be parallelized, the high computational load of neighborhood-based methods in comparison to other models is not trivial.

Cluster-based methods exhibit better scalability than neighborhood-based modeling, but there is a trade-off between prediction accuracy and scalability. These methods may not satisfactorily address the prediction for patients with rare conditions because they work based on predefined clusters. Especially in hierarchical clustering methods, in which final clusters are derived based on merging smaller clusters [53], the algorithm may fail to provide personalized predictions for patients with a rare condition.

Four studies embedded patient similarity analytics in their modeling approach even though they did not explicitly compute a patient similarity metric [27,32,36,42]. These studies reported improved prediction performance and overcame the limitations of neighborhood-based algorithms. However, these methods tend to be associated with increased computational and mathematical complexity. Mathematical complexity can lead to decreased interpretability in the context of how the model has learned to solve a problem. Nevertheless, neighborhood-based methods and cluster-based methods maintain a fair level of interpretability (a summary of the reviewed articles in terms of methodology is provided in Multimedia Appendix 2).

### Patient Similarity-Based Models Versus Conventional Models

Only two studies [39,40] directly compared the performances of patient similarity-based models and population-based models. Both demonstrated that patient similarity-based models resulted in better predictive performance. Lee et al [39] also compared the performance of patient similarity-based models to the Sequential Organ Failure Assessment [54] and the Simplified Acute Physiology Score [55], two widely used scoring systems in ICUs, and the patient similarity-based models showed a significant improvement.

### Gaps and Future Work

One of the factors that strongly affects predictive performance is the choice of predictors. Results show that researchers are searching for reliable predictors to enhance the performance of patient similarity-based models. In the context of personalized prediction models, the best possible predictors should have at least two characteristics: (1) be capable of capturing the progression of a patient’s health status, and (2) be as discriminative as possible. Applying TF-IDF technique could help boost the accuracy of similarity assessment for patients with rare conditions [21] in the predictor extraction phase because the IDF value is low for common clinical observations and high for rare observations. Although identifying the relevant predictors for patient similarity assessment is of special importance for precise prediction, only a few studies have considered this component in their proposed framework. Although feature selection techniques, predictor weighting schemes, and experts’ opinions were used in the reviewed articles to address this question, further studies are needed to identify appropriate predictors. However, as the number of predictors increases, the performance of many types of prediction models may decline and this can lead to a generalizability concern; hence, the need for external validation of prediction models. This challenge may be encountered in patient similarity predictive modeling, particularly with neighborhood-based methods where a model is developed from a small cohort of similar patients and the number of predictors may exceed the number of training instances. Therefore, creating a balance between the number of training instances—training sample size—and the number of predictors is important.

As observed in several studies, some values for a given patient may be missing. Although imputation methods can help deal with missing data, it is important to determine why the values are missing. Sometimes, associations exist between patterns of missing data and the outcomes. This type of information gap is referred to as informative missingness [56]. Further studies that account for this type of missingness are needed.

As mentioned previously, a wide variety of techniques have been employed in efforts to achieve personalized prediction. Neighborhood-based methods are among the most popular techniques. However, abundant room remains for progress in defining new patient similarity metrics. In addition, as suggested by Gottlieb et al [32], various similarity metrics based on different predictors can be combined to devise better similarity metrics.

There are some limitations to this review. First, although the article selection protocol was devised by all reviewers, there could have been a bias in selecting articles because title and abstract screening was done by only one reviewer. Second, the search process focused on the more generic terms covering the concept of EHR, and it might have excluded articles in which domain-specific words (eg, “diabetes data”) were used to describe the data source. Finally, due to inaccessibility to some EHR data in the included studies, data quality assessment was infeasible and all the studies received equal importance in the interpretation of the findings, which might have caused a bias in the results.

### Conclusion

Personalized medicine has the potential to facilitate predictive medicine, provide tailored prognoses/diagnoses, and prescribe more effective treatments. Interest is increasing in the use of personalized predictive modeling and various patient similarity-based models using EHRs have been described in the literature. This review has demonstrated the value of patient similarity-based models in critical health problems and noted the results of two studies [39,40] on the superiority of patient similarity-based models over population-based ones. The suggested future work could improve the capabilities of these models.

## Appendix

Multimedia Appendix 1 Search strings used to search databases.

Multimedia Appendix 2 Summary of the reviewed articles in terms of methodology (N=22).

## Abbreviations

APACHE-II: Acute Physiology and Chronic Health Evaluation II
ART: adaptive semisupervised recursive tree partitioning
CBR: case-based reasoning
DT: decision tree
EHR: electronic health data
ICU: intensive care unit
kd-tree: k dimensional tree
k-NN: k-nearest neighbor
LR: logistic regression
LSDA: locality sensitive discriminant analysis
LSR: local spline regression
PCA: principal component analysis
ROC: receiver operating characteristic
RSF: random survival forest
TF-IDF: term frequency–-inverse document frequency
