# Supplementary material for: Patient Similarity in Prediction Models Based on Health Data: A Scoping Review
Source: JMIR Med Inform. 2017 Mar 3;5(1):e7. doi: 10.2196/medinform.6730 (PMC5357318; doi:10.2196/medinform.6730)
Supplement: Multimedia Appendix 1 [file medinform_v5i1e7_app1.pdf]

Multimedia Appendix 1

Search Strings Used to Search Databases.

- *PubMed search strings*

((("predict*"[Title/Abstract] AND ("similarity based"[Title/Abstract] OR "similarity-based"[Title/Abstract] OR "similar patient"[Title/Abstract] OR "case specific"[Title/Abstract] OR "case-specific"[Title/Abstract] OR "Patient specific"[Title/Abstract] OR "patient-specific"[Title/Abstract] OR "participant specific"[Title/Abstract] OR "participant-specific"[Title/Abstract] OR "similar disease progression"[Title/Abstract] OR "similar record"[Title/Abstract] OR "similar trend"[Title/Abstract] OR "case-based"[Title/Abstract] OR "case based"[Title/Abstract] OR "similar case"[Title/Abstract] OR "similar clinical case"[Title/Abstract] OR "personalized"[Title/Abstract] OR "customized"[Title/Abstract] OR "patient level"[Title/Abstract] OR "tailor made"[Title/Abstract] OR "individualized"[Title/Abstract] OR "individualised"[Title/Abstract])) OR ("similarity metric"[Title/Abstract] OR "similarity measure"[Title/Abstract] OR "similarity score"[Title/Abstract] OR "similarity assessment"[Title/Abstract])) AND ("EHR"[Title/Abstract] OR "EMR"[Title/Abstract] OR "health record"[Title/Abstract] OR "health data"[Title/Abstract] OR "medical record"[Title/Abstract] OR "medical data"[Title/Abstract] OR "clinical data"[Title/Abstract] OR "clinical record"[Title/Abstract] OR "patient data"[Title/Abstract] OR "patient record"[Title/Abstract] OR "physiological data"[Title/Abstract] OR "longitudinal data"[Title/Abstract] OR "longitudinal record"[Title/Abstract] OR "MIMIC II"[Title/Abstract] OR "clinical database"[Title/Abstract]))

- *Scopus search strings.*

((((TITLE-ABS-KEY("predict*")) AND ((TITLE-ABS-KEY("patient similarit*" OR "participant similarit*" OR "temporal similarit*" OR "similarity based" OR "similarity-based" OR "similar patient" OR "similar participant" OR "case specific" OR "case-specific" OR "Patient specific" OR "patient-specific")) OR (TITLE-ABS-KEY("participant specific" OR "participant-specific" OR "similar disease progression" OR "similar record" OR "similar history record" OR "similar medical record" OR "EHR similarity" OR "similar trend" OR "case-based" OR "case based")) OR (TITLE-ABS-KEY("similar characteristic*" OR "similar case" OR "similar clinical case" OR "personalized" OR "customized" OR "patient level" OR "tailor made" OR "individualised" OR "individualized")))) OR (TITLE-ABS-KEY("similarity metric" OR "similarity measure" OR "similarity score" OR "similarity assessment"))) AND ((TITLE-ABS-KEY("EHR" OR "EMR" OR "health record" OR "health data" OR "medical record" OR "medical data" OR "clinical data" OR "clinical record" OR "patient data" OR "patient record" OR "physiological data" OR "physiological record" OR "longitudinal data")) OR (TITLE-ABS-KEY("longitudinal record" OR "MIMIC II" OR "clinical database")))) AND
(LIMIT-TO(LANGUAGE,"English"))

- *Web of Science search strings.*

(((TS=("predict*")) AND ((TS=("patient similarit*" OR "participant similarit*" OR "temporal similarit*" OR "similarity based" OR "similarity-based" OR "similar patient" OR "similar participant" OR "case specific" OR "case-specific" OR "Patient specific" OR "patient-specific")) OR (TS=("participant specific" OR "participant-specific" OR "similar disease progression" OR "similar record" OR "similar history record" OR "similar medical record" OR

"EHR similarity" OR "similar trend" OR "case-based" OR "case based")) OR (TS=("similar characteristic*" OR "similar case" OR "similar clinical case" OR "personalized" OR "customized" OR "patient level" OR "tailor made" OR "individualized" OR "individualised")))) OR (TS=("similarity metric" OR "similarity measure" OR "similarity score" OR "similarity assessment"))) AND ((TS=("EHR" OR "EMR" OR "health record" OR "health data" OR "medical record" OR "medical data" OR "clinical data" OR "clinical record" OR "patient data" OR "patient record" OR "physiological data" OR "physiological record" OR "longitudinal data")) OR (TS=("longitudinal record" OR "MIMIC II" OR "clinical database")))
