# Supplementary material for: Patient Similarity in Prediction Models Based on Health Data: A Scoping Review
Source: JMIR Med Inform. 2017 Mar 3;5(1):e7. doi: 10.2196/medinform.6730 (PMC5357318; doi:10.2196/medinform.6730)
Supplement: Multimedia Appendix 2 [file medinform_v5i1e7_app2.pdf]

Multimedia Appendix 2

Summary of the reviewed articles in terms of methodology (N=22).

**Authors**	**Similarity metric**	**Methodology**[a]	**Neighborhood-based**	**Cluster-based**	**Other algorithm**	**Programming language**[b]
Jurisica et al [16]	Context-based metric	1. Retrieving patients based on a context-based metric defined by a user.<br>2. Relaxing the context to retrieve more patients (optional).<br>3. Weighted aggregation of the retrieved outcomes is used for an index patient.	✓			NR
Bobrowski [17]	Euclidian distance	1. Designing a linear transformation by maximizing within-class compactness and between-class scatteredness.<br>2. Applying the transformation, and executing a *k*-NN (k=10) algorithm on the transformed data.	✓			NR
Park et al [19]	Euclidian distance	1. Learning the distribution of distances in the training set.<br>2. Finding the optimum cut-off probability using a grid search.<br>3. Retrieving similar patients within the distance threshold.<br>4. Weighted aggregation of the retrieved patients' outcomes is used for an index patient.	✓			JAVA

Authors	Similarity metric	Methodology[a]	Neighborhood-based	Cluster-based	Other algorithm	Programming language[b]
Saeed et al [21]	Correlation distance	1. Applying a discrete wavelet transformation.<br>2. Uniformly quantizing the wavelet coefficients' histogram at each scale to produce wavelet symbols.<br>3. Using IDF map of wavelet symbols as predictors.<br>4. Applying *k*-NN with majority vote within a threshold.	✓			NR
Chattopadhyay et al [23]	Absolute distance	1. Retrieving a cohort of patients who are similar to a new patient.<br>2. Splitting the retrieved data into cohorts with various risk levels.<br>3. Calculating the relative distance of a new patient from each cohort.<br>4. Assigning the new patient to the closest class.	✓			JAVA

**Authors**	**Similarity metric**	**Methodology**[a]	**Neighborhood-based**	**Cluster-based**	**Other algorithm**	**Programming language**[b]
Sun et al [24]	Mahalanobis Distance	1. Employing two schemes for deriving predictors:<br>a. Wavelet coefficients: applying a wavelet transformation and deriving top-10 wavelet coefficients.<br>b. Statistic predictors: calculating the mean and variance of two hours mean APB, systolic ABP, SPO_2 and heart rate data.<br>2. Defining a Mahalanobis distance by solving an optimization problem by minimizing the within-class squared distances and maximizing between-class squared distances.<br>3. Retrieving three most similar patients to an index patient based on the similarity metric learned in step 2.	✓			NR

Authors	Similarity metric	Methodology[a]	Neighborhood-based	Cluster-based	Other algorithm	Programming language[b]
Sun et al [25]	Mahalanobis Distance	1. Imputing the missing data:<br>a. Replacing with the mean.<br>b. Using the correlation between sensors and using linear regression imputation.<br>2. Applying wavelet transformation and deriving top-10 wavelet coefficients.<br>3. Defining a Mahalanobis distance by solving an optimization problem by minimizing the within-class squared distances and maximizing between-class squared distances.<br>4. Retrieving five most similar patients to an index patient based on the similarity metric learned in step two.	✓			NR

**Authors**	**Similarity metric**	**Methodology[a]**	**Neighborhood-based**	**Cluster-based**	**Other algorithm**	**Programming language[b]**
David et al [26]	Euclidian distance	1. Assigning random weights to the predictors.<br>2. Selecting neighbors for an index patient using Euclidian distance.<br>3. Applying singular value decomposition to map the data to a lower dimensional space.<br>4. Examining the discriminative power of the assigned weights.<br>5. Repeating steps (1)-(4) to achieve a set of random discriminative weights.<br>6. Combining all confirmed weighting vectors to derive a differential matrix.<br>7. Using the differential matrix to obtain the neighborhood of an index patient.<br>8. Using the weighted aggregation of the neighboring labels.	✓			NR
Houeland [27]	Tree-based metric	1. Generating a forest of randomly grown trees of height five.<br>2. Each tree sorts a patient into one of 16 leaf nodes (buckets).<br>3. Using *k*-NN (k = 1)—Two patients are said to be similar if they are in the same leaf node for a higher number of trees.	✓			NR

Authors	Similarity metric	Methodology[a]	Neighborhood-based	Cluster-based	Other algorithm	Programming language[b]
Wang et al [28]	Mahalanobis Distance	1. Learning a Mahalanobis distance for each party by maximizing inter-class compactness and between-class scatteredness.<br>2. Defining a quadratic optimization problem to derive a single optimal distance metric.<br>3. Using *k*-NN algorithm.	✓			NR
Wang et al [29]	Mahalanobis Distance	1. Learning a Mahalanobis distance using the following steps:<br>a. For each patient, retrieving *k* nearest neighboring cases based on the Euclidean distance.<br>b. Solving a local spline regression problem.<br>c. Aggregating local neighborhoods’ losses and minimizing the global loss of local spline regressions to derive the precision matrix.	✓			NR

Authors	Similarity metric	Methodology[a]	Neighborhood-based	Cluster-based	Other algorithm	Programming language[b]
		2. Using *k*-NN (*k* = 5) algorithm.				
Campillo-Gimenez et al [30]	XOR distance	1. Training an LR model on a part of the training set.<br>2. Using the LR coefficients and outcomes for assigning weights to the predictors and cases, respectively.<br>3. Exploiting a *k*-NN algorithm with an XOR patient similarity metric.	✓			R
Gottlieb et al [32]	Various similarity metrics	1. Calculating eight similarity metrics between hospitalizations and two similarity majors for ICD codes.<br>2. Combining the metrics into 16 hospitalization-discharge code associations.<br>3. Calculating the score of a potential discharge code for a new patient's hospitalization data.<br>4. Using an LR classifier to distinguish the true associations.			✓	MATLAB
Lowsky et al [33]	Mahalanobis distance	1. Defining a Mahalanobis distance with precision matrix equals to inverse of the covariance matrix of the training data.<br>2. Retrieving *k* most similar patients to a new patient.<br>3. Generating the Kaplan-Meier survival curve for the new patient based on	✓			MATLAB

Authors	Similarity metric	Methodology[a]	Neighborhood-based	Cluster-based	Other algorithm	Programming language[b]
		the retrieved cases.				
Hielscher et al [34]	Heterogeneous Euclidean Overlap Metric (HEOM) [27]	1. Splitting the dataset based on gender.<br>2. Selecting predictors by Correlation-based Feature Selection algorithm [28].<br>3. Employing *k*-NN with majority vote and weighting vote for classification	✓			NR
Zhang et al [36]	Jaccard similarity coefficient	1. Constructing a drug similarity matrix by using chemical structure extracted from PubChem, and drug target protein information extracted from DrugBank.<br>2. Building patient-drug similarity matrix using the Jaccard similarity coefficient between ICD9 diagnosis codes of patients and ICD9-format drug indications from MEDI database<br>3. Constructing patient similarity network using Jaccard similarity coefficient on ICD9 diagnosis codes.<br>4. Concatenating the three matrices and employing label propagation method to infer the efficiency of a drug for an index patient.			✓	NR
Henriques et al [37]	Coefficients' signs-based distance	1. Representing a signal by Haar wavelet coefficients.<br>2. Defining a similarity	✓			NR

Authors	Similarity metric	Methodology[a]	Neighborhood-based	Cluster-based	Other algorithm	Programming language[b]
		metric based on the sign of each coefficient.<br>3. Employing *k*-NN for prediction				
Lee et al [39]	Cosine similarity metric	1. Calculating all pair-wise cosine patient similarity metric between an index patient and patients in training set<br>2. Ranking the patients based on the similarity values<br>3. Using *k* most similar patients to train 3 prediction models:<br>a) Majority vote (*k*-NN)<br>b) LR<br>c) Decision tree	✓			R
Ng et al [40]	Mahalanobis distance	1. Supervised learning of a Mahalanobis patient similarity metric.<br>2. Computing patient similarity and identifying a cohort of *k* similar patients.<br>3. Selecting predictors.<br>4. Training an LR on the cohort for risk prediction.	✓			NR
Panahiazar et al [41]	Mahalanobis distance	1. Splitting patients based on their response to a medication (good or poor).<br>2. Clustering the patients using two approaches:		✓		NR

Authors	Similarity metric	Methodology[a]	Neighborhood-based	Cluster-based	Other algorithm	Programming language[b]
		a. Unsupervised clustering: *k*-means and hierarchical clustering.<br>b. Supervised clustering with medications as labels.<br>3. Measuring the similarity of an index patient to the mean of each cluster using a Mahalanobis distance.<br>4. Considering the medication of the most similar cluster for a new patient.				
Wang [42]	Tree-based distance	1. Constructing a tree by optimizing two-term objective function:<br>a. A human expert's knowledge term.<br>b. A data mining based term.<br>2. Using the tree to index patient profiles and then rapidly retrieve the nearest neighbors to a new patient.			✓	Python

Authors	Similarity metric	Methodology[a]	Neighborhood-based	Cluster-based	Other algorithm	Programming language[b]
Wang et al [43]	Euclidean Distance in a transformed space	1. Learning a transformed Euclidean distance by optimizing two-term objective function: a. Human expert's knowledge term. b. Data mining based term. 2. Using a *k*-NN algorithm.	✓			MATLAB

[a] Methodology: ABP: arterial blood pressure; ICD: International Classification of Diseases; IDF: inverse document frequency; k-NN: k-nearest neighbor; LR: logistic regression; SPO2: saturation of peripheral oxygen; XOR: exclusive or.

[b] NR: not reported.
